# Involuntary Psychiatric Hospitalization of Minors Due to Court Orders: Effectiveness Assessing Through a Case Series

**DOI:** 10.1192/j.eurpsy.2024.232

**Published:** 2024-08-27

**Authors:** E. Yerlikaya Oral, M. Tekden

**Affiliations:** ^1^Department of Child and Adolescent Psychiatry, University of Health Sciences, Bakirkoy Prof. Dr. Mazhar Osman Research and Training Hospital for Psychiatry, Neurology and Neurosurgery, Istanbul, Türkiye

## Abstract

**Introduction:**

Involuntary treatments in forensic psychiatry represents a complex intersection of mental health, legal systems and ethics. Judicial authorities may compulsorily refer children to inpatient clinics for receiving necessarily treatment. Despite its importance, there is limited research on the reasons behind and effectiveness of such interventions in minors.

**Objectives:**

The objectives of this study were to describe the clinical characteristics of minors who have risks of harming themselves and/or others so receiving involuntary treatment due to a court order. It is aimed to assess the effectiveness of involuntary treatment.

**Methods:**

A follow-up case series was conducted on 9 minors who hospitalized by court orders in a secure inpatient child and adolescent psychiatry clinic, in the year of 2023. Data collected from medical records, including demographic information, clinical presentation, diagnosis and discharge treatment. After one, three and six month of the discharge, interviews made with the patients and their families. Current data collected on treatment regimen, compliance, behavioral outcomes and reoffending rates. All data were anonymized to maintain patient confidentiality.

**Results:**

The case series consisted of 3 males and 6 females, with a mean age of 16.5 years at the time of admission. The most common reason to hospitalization was homicide risk 88%, followed by substance use 66%. Conduct Disorder was the most common diagnosis with the rate of 88%, followed by Substance Use Disorder(66%) and Attention Deficit and Hiperactivity Disorder(50%). 44% of minors had a history of juvenile delinquency. School dropout rates were 100%. Treatment consisted of a combination of individual and group therapy and medication. Treatment refusal rates were 88% so in terms of treatment, 88% of the minors in this sample treated with depot form antipsychotic medications, with the most common medication being risperidone. Overall all of the sample showed a significant reduction in disruptive behaviors during their hospital stay. Follow-up data collecting is still continue and preliminary statistics show us that relapse rates are low and treatment compliance is relatively high of the sample.

**Conclusions:**

The findings suggest that involuntary hospitalization can be effective in reducing disruptive behaviors and increasing treatment compliance in minors with conduct disorders, substance abuse disorders and a history of juvenile delinquency. These results underscore the need for comprehensive, multidisciplinary approaches that integrate psychiatric treatment, psychoeducation and social support. Given the relatively small sample size and short-term follow-up, further research is needed to determine the long-term effects of involuntary treatment and to identify factors that predict treatment response.

**Disclosure of Interest:**

None Declared

